# A Delayed Diagnosis of Hemorrhagic Shock in a Patient with Alcoholic Cirrhosis and Ascites on Bedside Ultrasound

**DOI:** 10.1155/2019/5895801

**Published:** 2019-12-10

**Authors:** Madeline Bach, Julian Choi, Rory A. Smith, Sarkis Arabian

**Affiliations:** Department of Internal Medicine, Arrowhead Regional Medical Center, Colton, CA, USA

## Abstract

Undifferentiated shock is a common and challenging problem in critical care. We present a case of hemorrhagic shock due to splenic and hepatic lacerations diagnosed by bedside paracentesis, initially misclassified as septic shock due to suspected spontaneous bacterial peritonitis (SBP). *Case*. A 47-year old man with a history of reported alcoholic cirrhosis and ongoing heavy alcohol use was brought to the emergency room after a syncopal event. He was found to be anemic (hemoglobin 9.9 g/dl) and hypotensive with a blood pressure of 64/34. Despite crystalloid infusion he remained hypotensive and required vasopressor support with norepinephrine. Bedside ultrasound revealed moderate ascites and as there was no evidence of active bleeding, his shock was attributed to sepsis due to SBP. A bedside paracentesis was performed which revealed gross blood. A repeat hemoglobin returned at 4.4 g/dl. Massive transfusion protocol was initiated and interventional radiology was emergently consulted due to concerns for intraabdominal hemorrhage; general surgery deemed the patient too unstable for surgical intervention. Angiogram revealed a splenic laceration and possible hepatic laceration, both embolized successfully. Internal medicine practitioners should keep the differential of hemorrhagic shock due to intraabdominal organ injury in mind for patients with undifferentiated shock.

## 1. Introduction

Undifferentiated shock in critically ill patients presents a complex problem. Bedside ultrasound is a useful tool for diagnosis in patients presenting with hypotension in the emergency department and in the intensive care unit (ICU) setting [1, 2]. The focused assessment with sonography for trauma (FAST) is an established method for detecting abdominal free fluid in trauma which is indicative of hemoperitoneum, an indirect sign of abdominal organ injury [3]. Unfortunately, ultrasound may not be able to differentiate between simple ascites in a patient with established cirrhosis or hemoperitoneum in the setting of undisclosed trauma. Furthermore, a patient in shock may not be stable for transfer for abdominal computed tomography (CT) imaging to evaluate for organ injury. We report a case of undifferentiated shock in a cirrhotic patient with unknown trauma which was initially diagnosed as septic shock due to SBP until a bedside paracentesis revealed hemoperitoneum, drastically shifting management and diagnosing life threatening hepatic and splenic lacerations.

## 2. Case Report

A 47-year-old man with a past medical history of alcoholic cirrhosis and ongoing alcohol abuse was brought to the emergency room after a syncopal episode. He complained of 7/10 epigastric pain associated with nonbloody emesis. He denied melena. He was tachycardic with a heart rate of 108 as well as hypotensive with a blood pressure of 64/34. His oxygen saturation was 96% on room air. He was alert with a Glasgow Coma Scale (GCS) of 14 but noted to be in severe distress, diaphoretic, and tremulous. His skin was cold and clammy and abdomen was noted to be distended and diffusely tender to palpation. A rectal exam was negative for gross blood. A FAST exam noted moderate ascites. A chest X-ray was performed and was negative for acute pathology.

Relevant laboratory data on admission included the following: white blood cell count of 7600 per mm^3^, bands of 38%, hemoglobin of 9.9 g/dl, platelet count of 149,000 per mm^3^, international normalized ratio (INR) of 2.5, aspartate aminotransferase (AST) of 213 U/liter, and alanine aminotransferase of 309 U/liter. Initial venous blood gas revealed a pH of 7.23, a venous bicarbonate level of 18 mmol/liter and a lactate of 6.62 mmol/liter.

Despite four liters of intravenous crystalloid fluid resuscitation the patient remained hypotensive at 75/58. Vasopressor support was initiated with intravenous norepinephrine titrated to a dose of 30 mcg/min and vasopressin at 0.04 units/min with minimal improvement in blood pressure to 94/76. Broad spectrum antibiotics consisting of vancomycin and piperacillin-tazobactam were administered due to concerns for septic shock. SBP was considered the cause of sepsis due to patient's abdominal pain, ascites, and bandemia.

The patient became increasingly more confused and agitated and the trachea was electively intubated for airway control. During this time, his abdomen was noted to be more distended. Patient was deemed too unstable for abdominal CT imaging as he was requiring multiple vasopressors. Postintubation he was transferred to the medical ICU for presumed septic shock.

On arrival to the ICU after initial stabilization of the patient a diagnostic paracentesis was performed in order to obtain culture for possible SBP. An appropriate pocket of fluid was visualized in the right lower abdominal quadrant. Approximately five ml of bloody ascites was aspirated. Concurrently, repeat lab results reported a hemoglobin level of 4.4 g/dl and an arterial blood gas with a lactate of 12.92 mmol/liter. General surgery was immediately consulted due to concerns for intraabdominal hemorrhage. At the same time the patient's girlfriend arrived to the hospital and stated that the patient had complained of falling in the bathroom earlier that day. General surgery recommended angiogram to locate the source of suspected intraabdominal bleeding. Interventional radiology was contacted for emergent angiogram to evaluate for a hepatic or splenic hemorrhage. Massive transfusion protocol was initiated and intravenous epinephrine was added to norepinephrine and vasopressin due to persistent hypotension. He was transferred to the IR suite.

**Figure 1:**
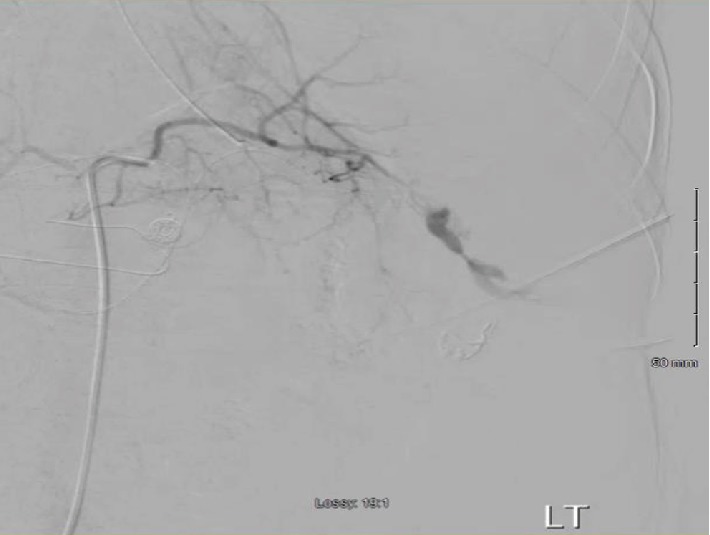
Angiogram of the spleen, showing distal splenic artery contrast extravasation.

**Figure 2:**
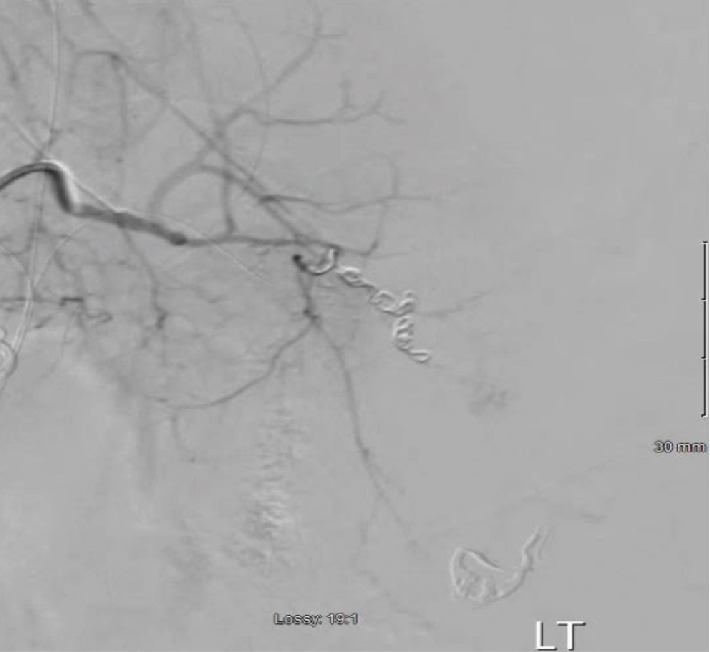
Angiogram, post embolization of the distal splenic artery with no evidence of extravasation.

The right common femoral artery was accessed and an angiogram was performed initially finding vague contrast blush in the right hepatic lobe which was empirically embolized with Gelfoam and detachable coils. There was extensive contrast extravasation from the distal splenic artery (Figure 1). The splenic artery was embolized and angiogram postembolization demonstrated no further contrast extravasation (Figure 2). Patient's blood pressure increased from 70 systolic to 100 immediately after embolization. On examination post angiogram, his abdomen was tense. Patient was also oliguric therefore, the decision was made to take him immediately for exploratory laparotomy for treatment of abdominal compartment syndrome. Exploratory laparotomy revealed a cirrhotic liver without signs of injury and a two-centimeter grade 1 splenic laceration at the lower pole which was not actively bleeding. A total of nine liters of bloody ascites were evacuated from the abdomen. The abdomen was left open and a Bogota bag was sutured to the fascia. He was transfused a total of 5 units of packed red blood cells, 4 units of fresh frozen plasma and a unit of platelets during the procedure.

He was brought back to the operating room (OR) two days later and was found to have active bleeding at the anterior surface of the hilum of the spleen. This was cauterized, stopping the bleeding. On hospital day four he was taken back to the OR and the abdomen was closed. He was extubated on hospital day seven. Hospital course was complicated by acute kidney injury which resolved, as well as seizures attributed to alcohol withdrawal treated with chlordiazepoxide. Patient was discharged home and at a clinic follow-up two months post hospitalization he was noted to be doing well and abstaining from alcohol.

## 3. Discussion

Undifferentiated shock is a frequent dilemma in critical care. Bedside ultrasound has become integral in the diagnosis of patients with undifferentiated shock and can substantially change the trajectory of treatment [1]. The abdominal FAST exam has proven invaluable in the management of hypotensive patients with blunt abdominal trauma; It has essentially replaced diagnostic peritoneal lavage as a quick and easy method of assessing whether there is free abdominal fluid indicative of hemoperitoneum [4, 5]. The role of the FAST exam in patients with underlying cirrhosis with ascites has not been studied as extensively. Our case was further complicated at admission by the unknown recent history of the patient falling at home, causing splenic injury and the hypotension being attributed to sepsis. The free fluid on ultrasound was thought to be simple ascites in a patient with known cirrhosis. The bloody ascites on paracentesis drastically changed our management.

Bloody ascites is associated with trauma in 18% of cirrhotic patients [6]. Akriviadis writes a traumatic cause of acute hemoperitoneum can be entirely unsuspected because history is commonly unreliable [7]. Cirrhotics who are actively drinking, such as in our case, can render the history inadequate. In addition, the risk of falls leading to intraabdominal trauma are likely increased in the alcoholic patient. The spleen is the organ most commonly injured by blunt abdominal trauma and abdominal CT scan is a highly reliable and accurate modality in diagnosing splenic injury [8]. An algorithm has been proposed which calls for an urgent abdominal CT scan in a patient with grossly bloody ascites found by paracentesis [7].

Unfortunately, our patient was too hemodynamically unstable for CT imaging.

The utility of emergent bedside paracentesis in hypotensive patients has been described previously. Blaivas reports a case of an alcoholic patient presenting to the ER with hypotension and no history of clear trauma who was found to have ascites on bedside ultrasound [9]. Similar to our case, the abdominal free fluid was initially attributed to simple ascites due to cirrhosis, however, when ultrasound guided paracentesis revealed blood, the patient was taken to the OR emergently and diagnosed with splenic rupture [9]. Blaivas writes that although ultrasound can identify fluid in the abdomen, it cannot identify the type of fluid. An emergent diagnostic paracentesis can lead to crucial changes in management, potentially decreasing patient morbidity [9].

Blunt abdominal trauma is not typically considered in the differential diagnosis for patients in shock admitted to a medical ICU. Cirrhotic patients may further complicate the clinical picture as free fluid visualized on ultrasound may be attributed to simple ascites rather than hemoperitoneum. Anemia in these patients may be attributed to a variceal bleed before considering intraabdominal organ injury. Our case illustrates the critical care practitioner should consider blunt abdominal trauma and intraabdominal injury as a cause of hemorrhagic shock in a patient with undifferentiated shock. Bedside ultrasound may reveal abdominal free fluid but it cannot characterize the type of fluid. Emergent bedside paracentesis in our hemodynamically unstable patient and the finding of bloody ascites drastically changed our management and led to surgical intervention and patient stabilization. Critical care specialists in a medical ICU should consider hemoperitoneum due to blunt abdominal injury in the unstable cirrhotic patient and not hesitate to perform emergent diagnostic paracentesis.
